# Novel variants provide differential stabilisation of human equilibrative nucleoside transporter 1 states

**DOI:** 10.3389/fmolb.2022.970391

**Published:** 2022-11-08

**Authors:** Jessica C. Boakes, Steven. P. D. Harborne, Jessie T. S. Ngo, Christos Pliotas, Adrian Goldman

**Affiliations:** ^1^ Astbury Centre for Structural Molecular Biology, School of Biomedical Sciences, University of Leeds, Leeds, United Kingdom; ^2^ Peak Proteins Ltd, Birchwood House, Larkwood Way, Tytherington Business Park, Macclesfield, United Kingdom; ^3^ MIBS, Biological and Environmental Sciences, University of Helsinki, Helsinki, Finland

**Keywords:** equilibrative nucleoside transporter (ENT), protein stabilisation, inhibition studies, mutagenesis, membrane protein

## Abstract

Human equilibrative nucleoside transporters represent a major pharmaceutical target for cardiac, cancer and viral therapies. Understanding the molecular basis for transport is crucial for the development of improved therapeutics through structure-based drug design. ENTs have been proposed to utilise an alternating access mechanism of action, similar to that of the major facilitator superfamily. However, ENTs lack functionally-essential features of that superfamily, suggesting that they may use a different transport mechanism. Understanding the molecular basis of their transport requires insight into diverse conformational states. Differences between intermediate states may be discrete and mediated by subtle gating interactions, such as salt bridges. We identified four variants of human equilibrative nucleoside transporter isoform 1 (hENT1) at the large intracellular loop (ICL6) and transmembrane helix 7 (TM7) that stabilise the *apo*-state (∆T_*m*_ 0.7–1.5°C). Furthermore, we showed that variants K263A (ICL6) and I282V (TM7) specifically stabilise the inhibitor-bound state of hENT1 (∆∆T_*m*_ 5.0 ± 1.7°C and 3.0 ± 1.8°C), supporting the role of ICL6 in hENT1 gating. Finally, we showed that, in comparison with wild type, variant T336A is *de*stabilised by nitrobenzylthioinosine (∆∆T_*m*_ -4.7 ± 1.1°C) and binds it seven times worse. This residue may help determine inhibitor and substrate sensitivity. Residue K263 is not present in the solved structures, highlighting the need for further structural data that include the loop regions.

## 1 Introduction

Equilibrative nucleoside transporters (ENTs, SLC29) are a major family of nucleoside transporters that play a crucial role in a complex array of nucleoside-related biological processes (Baldwin et al., 1999; Baldwin et al., 2004). ENTs regulate the transport of nucleosides, nucleobases, and nucleoside-derived therapeutics through energy-independent uniport. Their function is crucial in cells that lack the biopathways for *de novo* purine synthesis. Due to their biological importance, ENTs represent an important drug target (Baldwin et al., 1999; King et al., 2006). ENTs occur exclusively in eukaryotes and are expressed in most tissue types. There are four members of the human ENT family (hENT1-4), with each of the isoforms having different tissue expression, membrane localisation, substrate specificities and pharmacological properties (Jennings et al., 2001; Baldwin et al., 2005; Barnes et al., 2006; Young et al., 2008; Young et al., 2013).

hENT1 is the best characterized of the hENTs. It is the major nucleoside transporter present in plasma membranes and occurs in almost all tissue types but with varying abundance. It has a preferred specificity for adenosine, which is the most common and significant signalling molecule in purinergic pathways and contributes to vasodilation, inflammation, and neuromodulation (Dunwiddie and Masino, 2001; Molina-Arcas et al., 2008; Parkinson et al., 2011; Pedata et al., 2016). Due to its prevalence and substrate preference, hENT1 targeting is an important therapeutic approach for many pathologies. hENT1 is the target of several important pharmaceutical agents such as adenosine reuptake inhibitors, which are commonly used in cardiac therapies, and DNA replication terminating nucleoside analogues, as used in anticancer and antiviral therapies (Sirotnak and Barrueco, 1987; Sundaram et al., 1998; Sundaram et al., 2001; Huang et al., 2017). Furthermore, high levels of hENT1 expressed in cholangiocarcinomas are correlated with increased survival rates following treatment with the anticancer therapeutic gemcitabine (Pastor-Anglada and Pérez-Torras, 2015; Verdaguer et al., 2017; Tavolari et al., 2019). Chemotherapeutic resistance to anticancer and antiviral therapies remains a significant challenge. The development of new therapeutics is essential in overcoming chemotherapeutic resistance (Amrutkar and Gladhaug, 2017). However, previous studies suggest ENTs have poor tolerance for chemical diversity in the type of interactions that lead to the high affinity binding of ligands (Playa et al., 2014; Rehan et al., 2015). Thus, the development of new and clinically relevant drugs presents additional challenges. Structure-based drug design is crucial for improving therapeutic efficacy (Baldwin et al., 1999; King et al., 2006; Lapponi et al., 2016).

In 2019 the first (and to date, the only) two structures of an ENT were reported, solved by X-ray crystallography. Wright and Lee, (2019) determined the structures of mutated, ICL6-loop truncated hENT1 with two chemically distinct inhibitors bound, nitrobenzylthioinosine (NBMPR) (PDB: 6OB6) and dilazep (PDB:6OB7), both in an outward facing conformation. This arrangement in a distinct outward facing conformation suggests that ENTs utilise an alternating access mechanism of action, as is seen in MFS transporters. MFS transporters have 12 TMs that are arranged as N- and C-terminal domains, TM1-6 and TM7-12 respectively (Supplementary Figure S1). In the alternating access model these domains undergo large conformational rearrangements to adopt one of two major alternating conformations, inward-facing (Ci) and outward-facing (Co) (Drew et al., 2021). Transport cycle transitions require the occupation of a series of discrete intermediate states (Law et al., 2008; Quistgaard et al., 2016). Gating interactions, such as salt bridges, may help distinguish different intermediate states and have been shown to be sites of function and regulation (Chakrapani, 2015; Fowler et al., 2015). The structure in which NBMPR is bound has gating interactions at the extracellular region of the central cavity, thereby representing an intermediate occluded outward-facing state (Wright and Lee, 2019). However, to understand the mechanism fully, it is important to stabilise full-length transporter in different states. We have used the program IMPROvER for rational design of stabilising mutations in integral membrane proteins including hENT1 (Harborne et al., 2020).

ENTs have 11 TMs arranged in asymmetric N- and C-terminal domains, TM1-6 and TM7-11, respectively. The X-ray structures of hENT1 identified that, as predicted (Valdés et al., 2009), the fold of hENT1 matches that of TM1-11 in MFS transporters (Supplementary Figure S1). However, as TM12 is missing, the TM9 of hENT1 is arranged to occupy the space that is occupied by TM9 and TM12 in MFS transporters (Wright and Lee, 2019). In addition to the missing TM12, ENTs lack the canonical MFS A-motif. The A-motif in MFS transporters is located at the intracellular loop between TM2 and TM3 (and/or TM8 and TM9) and is essential for the transport activity in many MFS transporters. Interactions between the A-motif and TMs provide conformational stabilisation and contribute to the intracellular gating mechanisms of MFS transport in various conformational states (Jessen-Marshall et al., 1995; Radestock and Forrest, 2011; Quistgaard et al., 2016). Instead of an A-motif, ENTs feature an extensive network of hydrophobic contacts in the intracellular regions of TM4, TM5, TM10 and TM11 (Wright and Lee, 2019). These hydrophobic contacts, along with additional highly conserved polar and charged interactions, contribute to the intracellular gating mechanisms of hENT1 (Wright and Lee, 2019). These structural differences between MFS and ENTs support the suggestion that ENTs utilise a mechanism of action that is distinct from that in MFS transporters (Supplementary Figure S1).

hENT1 features two distinct loop regions: the extracellular loop that connects TM1 and TM2 (ECL1), and the large intracellular loop between TM6 and TM7 (ICL6). N-linked glycosylation of N48 in the ECL1 of hENT1 has been shown to be critical for NBMPR sensitivity and substrate transport efficiency (Bicket and Coe, 2016). ICL6 appears not to be essential for transport activity but instead contributes to the fine-tuning of transport regulation, possibly through interactions with other proteins (Reyes et al., 2011a; Reyes et al., 2011b; Aseervatham et al., 2015). For instance, phosphorylation of ICL6 by protein kinase A or protein kinase C has been shown to modulate ENT1 transport (Hughes et al., 2015). Both ECL1 and ICL6 are absent from the available structures. The absence of ECL1 is likely due to poor resolution in this region due to intrinsic disorder. However, the deletion of the ICL6 region (Δ243-274), in addition to the introduction of three stabilising point mutations, was required to generate a construct amenable to crystallisation (Wright and Lee, 2019). Here we present investigations into variants of hENT1 for the stabilisation of apo-hENT1. In addition, we present further investigations into residues specific for the stabilisation of the NBMPR-bound state of hENT1, which supports the role of ICL6 in the regulation of hENT1.

Our methodology for the work on hENT1 utilises the well-established but multi-step Bac-to-Bac™ (Invitrogen) expression system in *Spodoptera frugiperda* (Sf9) cells. For technical reasons, we decided to sequence baculovirus shuttle vector (bacmid) DNA. Here, we report a new method for the extraction of bacmid DNA from Sf9 cells for subsequent PCR amplification and Sanger sequencing. This allowed us to interrogate and validate DNA sequences at experimental ‘end-points’.

## 2 Materials and methods

### 2.1 Stability and inhibitor assays

#### 2.1.1 Expression cultures and solubilisation

Expression of hENT1 variants identified by IMPROvER (Harborne et al., 2020) was achieved using the Bac-to-Bac® (Invitrogen) expression system in Sf9 cells. Expression cultures were set up using fresh mid-log phase Sf9 cells, diluted to a density of approximately 1.0 x 10^6^ cells/mL in 15 ml pre-warmed Insect-XPRESS™ Protein-free Insect Cell Medium (Lonza). Cultures were set up in 50 ml sterile culture vessels. The preparation and amplification of baculovirus has been described previously (Harborne et al., 2020). Using either 750 µl of the first virus amplification, 500 µl of the second amplification, or 250 µl of the third amplification, baculovirus was added to begin infection and expression of hENT1. Cultures were incubated at 27°C for 3 days with shaking on an orbital shaker at 270 rpm. Cells were then harvested by centrifugation at (250 x *g*, 20°C, 10 min) and stored at -80°C until use. Expression cultures were performed in four biological repeats.

Three cell pellets for each variant were thawed on ice, and each pellet resuspended in resuspension buffer (phosphate buffered saline pH 7.4, EDTA-free protease inhibitor cocktail, 0 or 20 µM NBMPR) to a final volume of 500 μl. Cell resuspensions were solubilised by the addition of 1% (w/v) n-dodecyl-β-D-maltoside (DDM) and incubated with end over end turning for 1 h at 4°C. Solubilised protein was then isolated by centrifugation (20,817 x *g*, 4°C, 1 h). GFP-linked hENT1 expression and solubilisation was confirmed by measuring the supernatant in a QFX fluorometer (DeNovix) and deducting the background, determined by a hENT1-negative cell control.

#### 2.1.2 Stability assay

10 x 50 μl aliquots of supernatant were prepared, one aliquot was retained on ice (4°C) while the remaining nine aliquots were incubated in a T100 thermal cycler (Bio-Rad) at a single temperature (30, 35, 40, 45, 50, 55, 60, 65 or 70°C) for 10 min, followed by a 10-min incubation at 4°C. Following heating, precipitated protein was removed by centrifugation (20,817 x *g*, 1 h, 4°C). 40 μl of each supernatant was then transferred to a fresh well of a 96-well PCR plate and 10 μl of 5x SDS loading dye added. 15 μl of each sample was applied to a 4–20% Mini-PROTEAN™ TGX PreCast Gel (Bio-Rad) and run at 150 V for 1 h. In gel hENT1-linked GFP fluorescence was visualised using either a G:BOX (G:BOX Chemi XX6 with Blue LEDs; Syngene) or an iBright FL 1500 (ThermoFisher) Scientific (Supplementary Figure S2). GFP signal intensity of GFP-linked hENT1 bands were quantified using ImageJ. In all experiments using GFP-tagged membrane proteins, there is always a significant fraction of GFP without the membrane protein attached, presumably due to in-cell degradation/truncation. We used in-gel fluorescence to be able to separate this soluble GFP signal from that covalently attached to hENT.

#### 2.1.3 Data-fitting and statistical analysis

The *T*_*m*_ of hENT1 in each condition was obtained by plotting the fluorescence signal of each temperature point after normalisation to the on-ice sample. Data were fit with a four-parameter dose-response curve (variable slope) by non-linear least-squares fitting in GraphPad Prism 9.0. The inflection point of the fitted curve represents the temperature at which half of the protein is denatured and is assigned as *T*_*m*_. Δ*T*_*m*_ was calculated by subtracting the relevant negative control *T*_*m*_ value. The standard error of the mean (SEM) was calculated for all values, with error propagation factored in for Δ*T*_*m*_ using the following equation:

$${SEM}_{\Delta T_{m}}=\sqrt{{\left({SEM}_{\Delta T_{m-ve\;control}}\right)}^{2}+{\left({SEM}_{\Delta T_{m}}\right)}^{2}}$$

Statistical analysis was performed using ordinary one-way ANOVA with a Dunnet follow-up test for multiple comparisons in GraphPad Prism 9.0.

#### 2.1.4 Inhibitor binding assay

A working stock of [Benzyl-3H]-nitrobenzylthioinosine (PerkinElmer) ([3H]-NBMPR) was prepared by diluting stock [3H]-NBMPR to a final concentration of 2 µCi ml^−1^ (64 nM). A working stock of dipyridamole (1 mM) was prepared in DMSO. Single cell pellets for the variants of interest were resuspended in resuspension buffer (phosphate buffered saline pH 7.4 and EDTA-free protease inhibitor cocktail) to a final volume of 500 µl. The optical density of each cell resuspension was measured at 600 nm to inform normalisation. The cell resuspensions were divided into ten 50 µl aliquots for three technical repeats of three assay conditions: (1) no inhibitor (-/-), (2) 32 nM [3H]-NBMPR (+/-) and (3) 32 nM [3H]-NBMPR and 20 µM dipyridamole (+/+). The remaining samples were applied to SDS-PAGE gel and analysed for total in-gel GFP fluorescence for each hENT1 variant, as described in “Stability assay”.

All conditions were incubated at room temperature for 1 h. Samples were applied to a GF/B filter (Whatman) pre-equilibrated in washing buffer (phosphate buffered saline pH 7.4) on a vacuum manifold (Promega). The liquid was pulled through the filters under vacuum and washed three times with 2 ml of washing buffer. Filters were incubated overnight at room temperature in 10 ml of Ultima Gold scintillant (PerkinElmer). Radioactive disintegrations from bound [3H]-NBMPR in samples were quantified in counts per minute using a TriCarb scintillation counter (PerkinElmer) using 10-min reads, which were performed twice. Background (−/−) and non-specific binding (+/+) were subtracted from the [3H]-NBMPR (+/-) values to determine hENT1 variant specific binding. The specific radioactive signal was normalised for each sample against the intensity of the in-gel GFP-linked band for each corresponding hENT1 variant. These normalised values were then scaled relative to wild type values.

### 2.2 Extraction and analysis of bacmid DNA from Sf9 cells

#### 2.2.1 DNA extraction

DNA was extracted from both whole and insoluble fractions of Sf9 cells using a NucleoSpin Plasmid isolation mini kit (Macherey-Nagel) following the manufacturer’s protocol for plasmid DNA isolation of low-copy plasmids from *Escherichia coli*. However, where manufacturer’s guidelines suggest use of 5–10 ml of a saturated *E. coli* Luria broth (LB) culture, we substituted ‘1.0 x 10^6^ cells/mL of Sf9 culture’ for 1 ml saturated *E. coli* culture. In this study, 15 ml Sf9 cultures at 1.0 x 10^6^ cells/mL were processed as equivalent to 15 ml saturated *E. coli* cultures. 5–15 ml volumes of Sf9 cells were used with success. The pellet achieved following lysis is characteristically more glutinous than is seen with *E. coli* and therefore requires additional care during aspiration. Increases to spin durations (11,000 *x g,* 10–20 min) and/or repeat spins (2-3 x 11,000 *x g*, 5–10 min) are well tolerated and may be beneficial for ease of aspirating the lysate. We sent this initially-extracted bacmid DNA for sequencing as per standard protocols. However, this was unsuccessful and resulted in low signal to noise ratios and overlapping peaks in chromatograms. This is possibly due to mixed DNA present in the samples or packing of the DNA in a way that was incompatible with the techniques used (McCarthy and Romanowski, 2008). We therefore decided to PCR-amplify the extracted bacmid DNA before sequencing.

#### 2.2.2 DNA amplification

The construct used for the expression of hENT1 variants features a C-terminal TEV-GFP-His^8^ and was originally cloned into a pFastBac^™^ 1 Expression Vector (Invitrogen). PCR amplification of the full GFP-tagged hENT1 construct was performed using a forward primer that anneals downstream of the polyhedrin promoter of the pFastBac™ vector (5′-GGA​TTA​TTC​ATA​CCG​TCC​CA-3′), and a reverse primer which anneals to the 5′ region of the SV40 PolyA sequence (5′- CAA​ATG​TGG​TAT​GGC​TGA​TT-3′). Therefore, amplification with these two primers provides confirmation of pFastBac™ recombination in the extracted DNA, and an amplification product of ∼2.3 Kbp suggests that the target insert is present. A PCR reaction was prepared for each pellet DNA prep with 5 μl 2 x Q5 polymerase pre-mix (New England BioLabs (NEB)), 1 ng μl^−1^ template bacmid DNA, 0.5 μM forward primer, 0.5 μM reverse primer and made up to a final volume of 10 μl with ultra-pure H_2_O. The PCR reaction was performed using a T100 thermal cycler (Bio-Rad) with the following programme: initial denaturation at 98°C for 7 min, before performing 30 cycles of 30 s denaturation at 98°C, 30 s annealing at 61°C and 2 min of extension at 72°C, followed by a final extension of 5 min at 72°C. Amplification of the insert was confirmed by agarose gel electrophoresis. Samples were prepared with 1 μl PCR product, 1 x DNA loading dye (NEB) and made up to a final volume of 6 μl by the addition of ultra-pure H_2_O and applied to an agarose gel (1% (w/v) agarose, 1 x TAE, 0.5 x SYBR Safe). Agarose gels were imaged using a G:BOX (SynGene).

#### 2.2.3 DNA sequencing

PCR clean-up was performed using the NucleoSpin Gel and PCR Clean Up Kit (Macherey-Nagel) and the final DNA products were sent for DNA sequencing (Eurofins). Sequencing was performed using the same forward primer used in amplification to provide N-terminal coverage of hENT1. Additional sequencing was performed with a reverse primer which anneals to the N-terminus of the GFP, thereby providing C-terminal coverage of hENT1 only. In combination these two primers provide coverage of the full-length hENT1. Sequencing was analysed using GeniousPrime 2021.2.2.

## 3 Results

### 3.1 Validation of variant identity through the sequencing of bacmid DNA

During our investigation of hENT1 variants that we had earlier predicted as stabilising using our program IMPROvER (Harborne et al., 2020)**,** we discovered some inconsistencies in the identity of the gene products being expressed by the Sf9 insect cells. We therefore decided to re-sequence from the bacmid DNA products, to confirm that the sequence and protein matched. We found that Sanger sequencing of bacmid DNA produced by the insect cells was preferable to sequencing of the preceding shuttle plasmid produced in *E. coli* due to potential handling errors. Thus, we developed an easy and robust method for isolating and sequencing the bacmid DNA.

Bacmid DNA was successfully isolated from both whole cells and the insoluble fractions following protein solubilisation but could not be sequenced. However, bacmid DNA extracted from both whole cells and insoluble fractions was amenable to PCR amplification. PCR amplification and sequence determination by Sanger sequencing (Eurofins) was successful for all 48 variants, although some variants required several rounds of sequencing before the mutation was identified, because the quality of the initial DNA from the bacmid preparation was variable, possibly due to the nature of the pellet (see Materials and Methods). Of the 48 bacmids sequenced, we found 20 unique variants (inclusive of wild type) (S1 Table) in 3–15 repeats, due to the inconsistencies initially observed. Where repeats of variants were identified the data were combined, thus accounting for the variance of the n value.

### 3.2 Comparing the stability of hENT1 variants between apo and inhibitor-bound states

#### 3.2.1 Wild type hENT1 and identifying variants of interest

**FIGURE 1:**
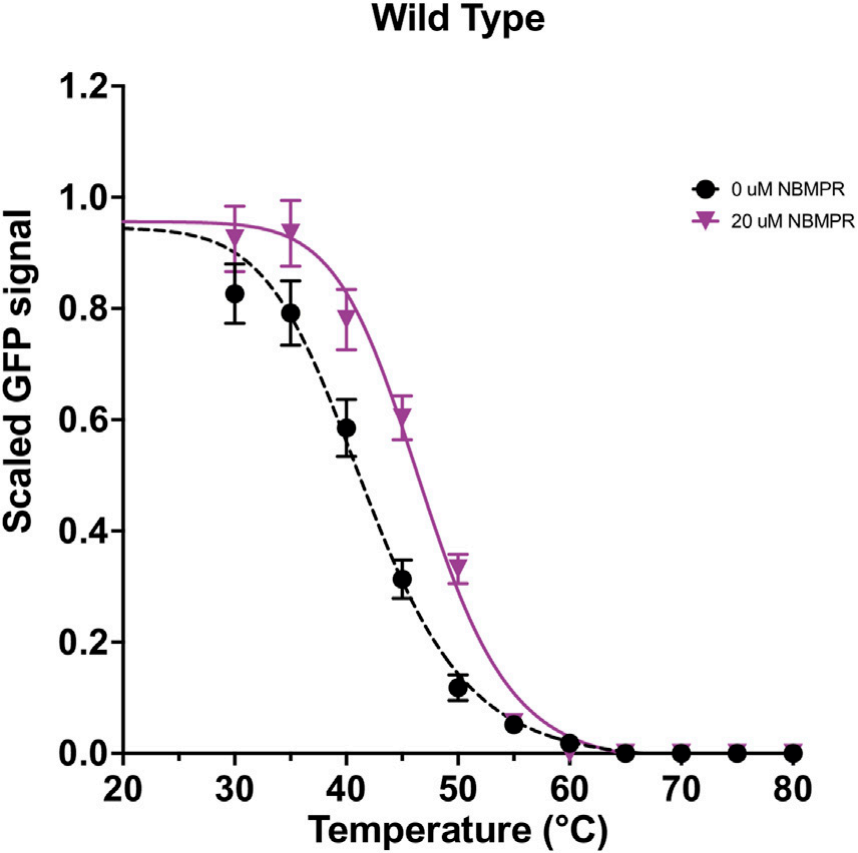
Stability curves for wild type hENT1 in both *apo* and NBMPR-bound states. Wild type *apo*-state and NBMPR curves were collected as an average of at least three repeats. Error bars are representative of SEM. Data were fit with a four-parameter dose-response curve (variable slope) by non-linear least-squares fitting in GraphPad Prism 9.0.

**FIGURE 2:**
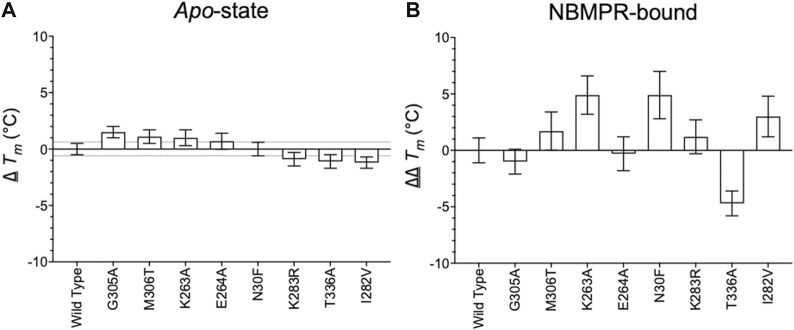
Relation of *apo* and NBMPR-bound hENT1 variants to wild-type. **(A)** ∆ *T*_*m*_ of all hENT1 variants in an *apo*-state, collected as an average of at least 3 repeats. The upper and lower bounds of the 0.6°C cut-off, as calculated by average SEM ∆*T*_*m*_, is represented by the dotted line. **(B)** ∆∆ *T*_*m*_ of all hENT1 variants in an NBMPR-bound state, collected as an average of at least three repeats. Error bars are representative of error propagation as detailed in “Methods and materials: Data-fitting” section. Please see Supplementary Figure S1 for full curves of *apo*-hENT1 variants.

The *T*_*m*_ determined for *apo* wild type hENT1 was 42.0 ± 0.3°C (Figure 1). Using a 0.6°C cut-off (average SEM ∆*T*_*m*_), four hENT1 variants were identified as stabilising, one additional variant was identified as neutral, and the remaining 14 variants were destabilising (Figure 2A, Supplementary Figure S3 and Supplementary Table S1). Stabilising, neutral and three of five variants that were not statistically significant destabilisers (G305A, M306T, K263A, E264A, N30F, K283R, T336A, I282V) were selected for further investigation (Supplementary Figure S4 and Supplementary Table S1). Stability assays in the presence of 20 µM NBMPR were performed on these eight variants and *wt* to investigate differences in the stabilising effects between the *apo* and NBMPR-bound states (Figure 2B). The ∆*T*_*m*_ determined for NBMPR-bound vs *apo* wild type hENT1 was 5.0 ± 0.8°C (Figure 1). Using a 5.0 ± 1.3°C cut-off (wild type ∆ *T*_*m*_ ± average SEM ∆ *T*_*m*_) four hENT1 variants (K263A, N30F, I282V, M306T) were identified as having an increased stabilising effect on the NBMPR-bound state, three variants were neutral (K283R, G305A, E264A), and one variant was destabilising (T336A) (Figure 2B and Supplementary Table S2).

#### 3.2.2 Mutation of TM1 and the central cavity

**FIGURE 3:**
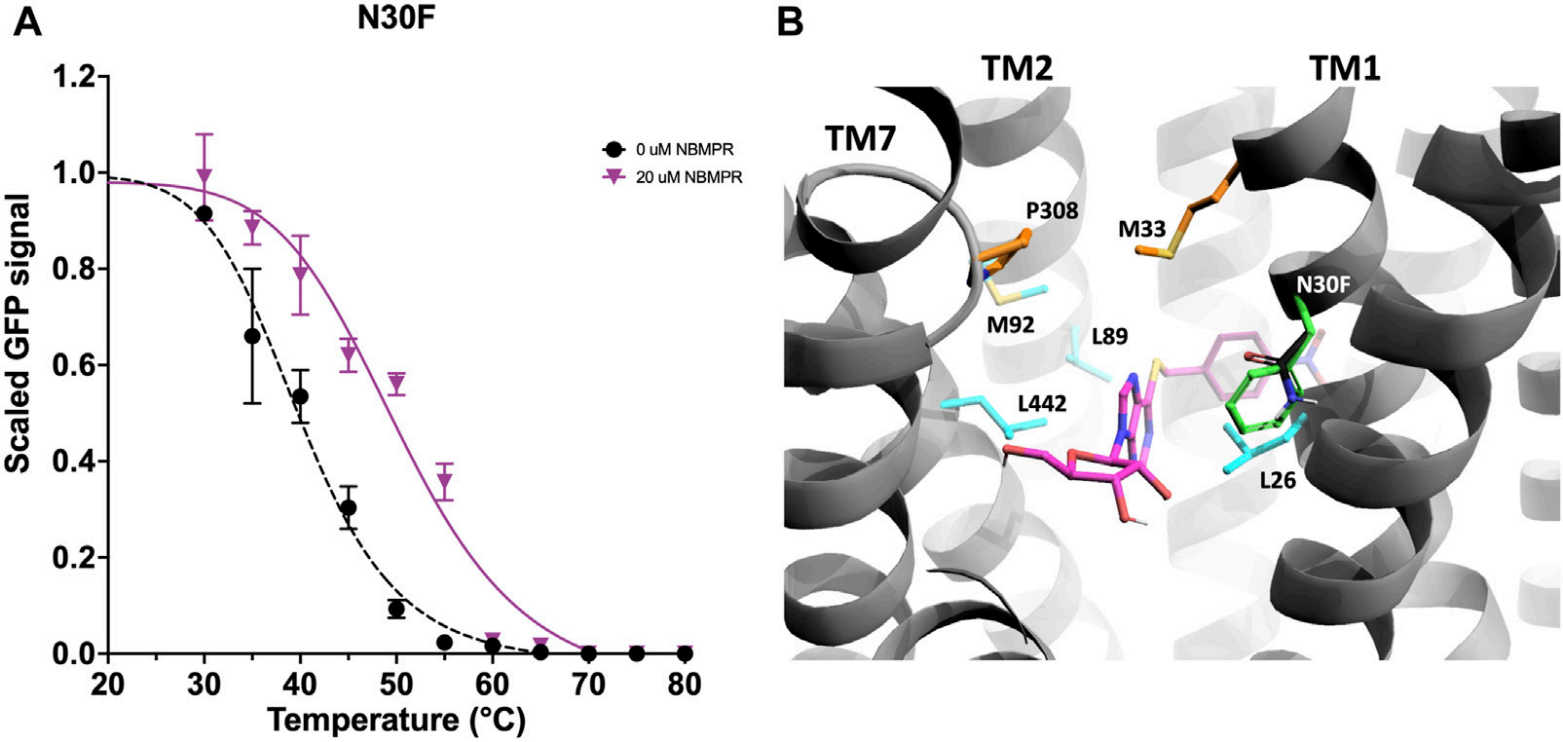
Investigation and rationalisation of variant N30F stabilisation of the NBMPR-bound state. **(A)** Data generation, fitting and error analysis performed as detailed in wild type. N30F *apo*-state curves were collected as an average of 10 repeats, whereas NBMPR-bound curves were collected as an average of 3 repeats. **(B)** A close-up view into the central cavity of hENT1 withTM8 removed for clarity. Side chains of residues involved in the surrounding of the purine moiety of NBMPR (pink) are shown in cyan. Residues involved in formation of the extracellular thin gate are shown in orange. Native N30 is shown in black, and variant N30F in green.

Variant N30F, which is located on TM1, has a neutral effect on the *apo*-state, ∆ *T*_*m*_ of 0.0 ± 0.6°C (Figure 2A, Supplementary Figure S3 and Supplementary Table S1). However, this mutation provides considerable stabilisation to the NBMPR-bound state, ∆∆*T*_*m*_ 4.9 ± 2.1°C (Figure 2B, Figure 3A, Supplementary Figure S4 and Supplementary Table S2). Therefore, N30F has an *apo*-neutral but NBMPR-bound stabilising effect. In the NBMPR-bound structure (PDB: 6OB6), interactions at TM1 are seen to contribute to both inhibitor binding and the gating mechanism which represents the occluded state in the outward-facing conformation (Wright and Lee, 2019). The hydrophobic residues L26 on TM1, along with M89 and L92 on TM2, and L442 on TM11 are shown to surround the purine moiety of NBMPR. M33 on TM1 and P308 on TM7 form a narrow constriction point which prevents the NBMPR from releasing freely into the extracellular side (Figure 3B), thereby forming extracellular gating interactions (Wright and Lee, 2019).

N30 faces into the central cavity and sits one helix turn above L26, and 0.75 helix turn below M33 (Figure 3B). Therefore, N30F may contribute to specific stabilisation of the NBMPR-bound state in several ways. Substitution of a polar side chain to a bulky, hydrophobic side chain increases the overall hydrophobicity of the central cavity and may support the hydrophobic environment around the NBMPR purine moiety. Additionally, the aromatic ring of the phenylalanine may contribute to π-π stacking interactions with the purine moiety. Furthermore, the bulky substitution of phenylalanine may protrude into the central cavity and therefore, may support the occlusion at the extracellular side established by the gating interactions between M33 (TM1) and P308 (TM8).

#### 3.2.3 Mutation of TM7

**FIGURE 4:**
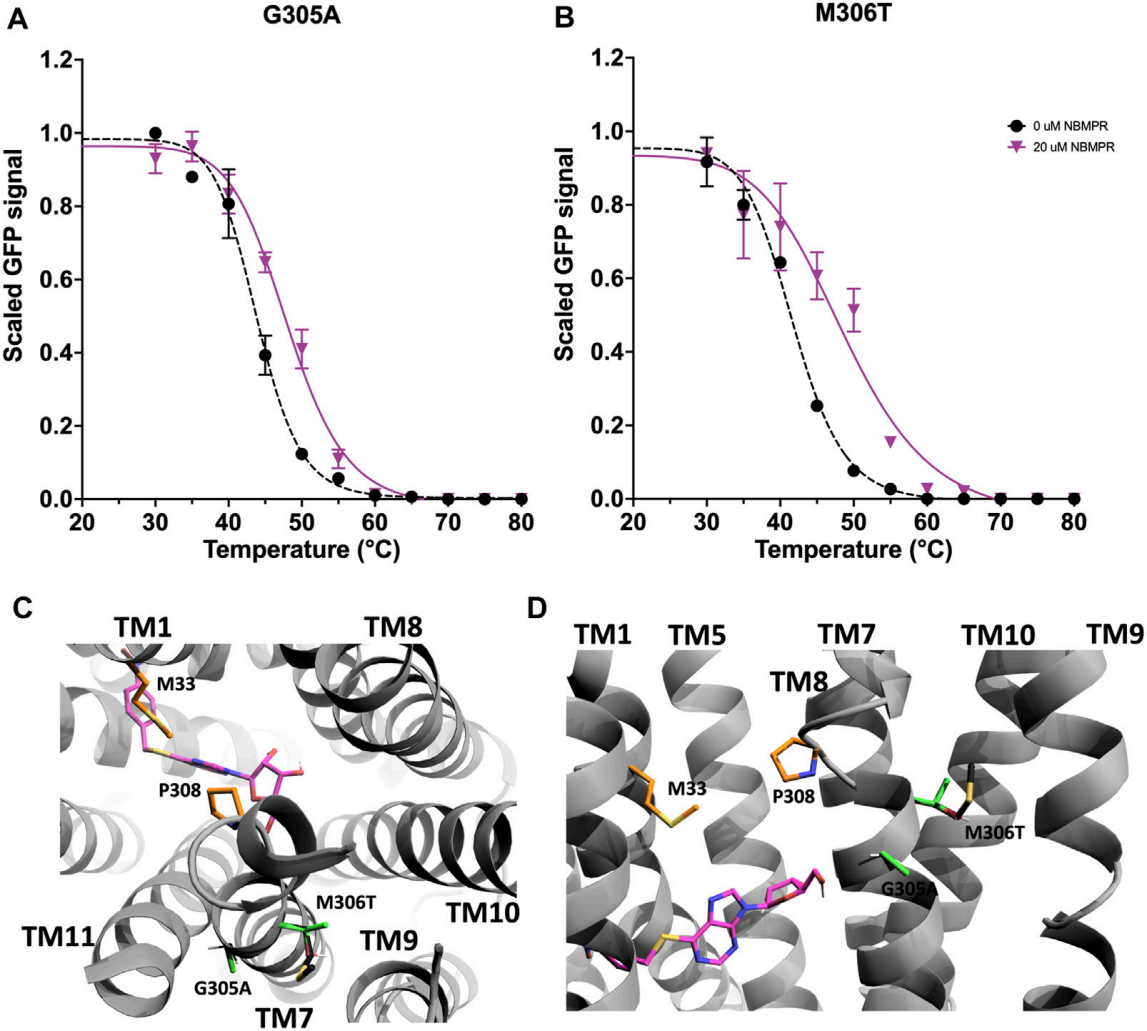
Investigation and rationalisation of variants G305A and M306T stabilisation of hENT1. Data generation, fitting and error analysis performed as detailed in wild type. **(A)** G305A *apo*-state curves were collected as an average of 8 repeats, whereas NBMPR-bound curves were collected as an average of 3 repeats. **(B)** M306T *apo*-state curves were collected as an average of 5 repeats, whereas NBMPR-bound curves were collected as an average of 3 repeats. **(C)** A top-down and **(D)** a close-up perpendicular view into the central cavity of hENT1 (TM11 was removed in D for clarity). NBMPR is shown in pink. Residues involved in formation of the extracellular thin gate are shown in orange. Native residues are shown in black, and mutations are shown in green.

G305 and M306 are located on the extracellular domain of TM7 and face towards TM11 and TM9, respectively (Figures 4C,D). Individual mutations at these residues, G305A and M306T, stabilise the *apo*-state by 1.5 ± 0.5°C and 1.1 ± 0.6°C (Figure 2A, Supplementary Figure S3 and Supplementary Table S1). Additionally, both variants result in stabilisation in the NBMPR-bound state that is similar to that seen in wild-type, ∆∆*T*_*m*_ -1.0 ± 1.1°C and 1.7 ± 1.7°C, respectively (Figure 2B, Figure 4A, Figure 4B and Supplementary Table S2). It is possible that both G305A and M306T stabilise the *apo*-state by improving the helix-packing interactions between TM11 and TM9.

**FIGURE 5:**
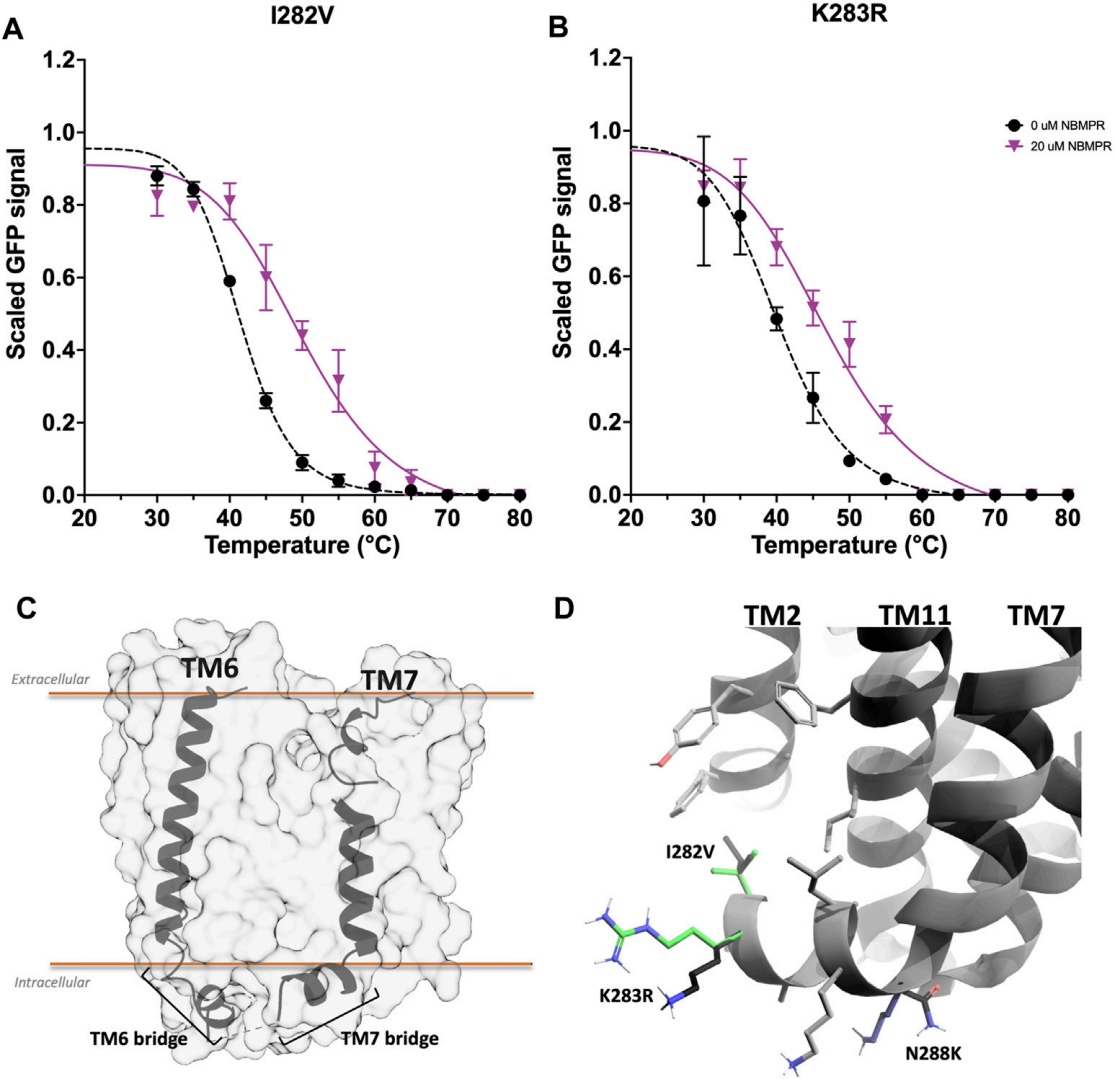
Investigation and rationalisation of variants I282V and K283R stabilisation of hENT1. Data generation, fitting and error analysis performed as detailed in wild type. **(A)** I282V *apo*-state curves were collected as an average of 12 repeats, whereas NBMPR-bound curves were collected as an average of 3 repeats. **(B)** K283R *apo*-state curves were collected as an average of 12 repeats, whereas NBMPR-bound curves were collected as an average of 3 repeats. **(C)** A perpendicular view of TM6 and TM7. TM1-5 and TM8-11 removed for clarity. **(D)** A close-up perpendicular view of the intracellular region of the TM7 bridge of hENT1. The side chains of neighbouring native residues are shown in grey. Mutations I282V and K283R are shown in green. The intracellular kink of TM7 is also the location of one of the three stabilising mutations (N288K, shown in blue) that was introduced by Wright & Lee to generate a crystallisable hENT1 construct (Wright and Lee, 2019). Native residues of all mutations are shown in black.

Variants I282V and K283R are also located on TM7. There is a kink in the intracellular region of both TM6 and TM7 that results in a short transverse helix that bridges the connection between the ICL and the TMs (Figure 5C). I282V and K283R are located at this bridge region of TM7. K283 faces towards the cytosol and I282 faces towards a hydrophobic region of TM2 and TM11 (Figure 5D). In the *apo*-state both I282V and K283R are destabilising, ∆*T*_*m*_ -1.2 ± 0.5°C and -0.9 ± 0.6°C, respectively (Figures 2A, Supplementary Figure S3 and Supplementary Table S1). However, I282V stabilises the NBMPR-bound state more than wild type, ∆∆*T*_*m*_ 3.0 ± 1.8°C (Figure 2B, Figures 5A, Supplementary Table S2), thus has an *apo*-destabilising but NBMPR-bound stabilising effect. This mutation may contribute to NBMPR-bound state stabilisation through tightening of hydrophobic interactions within this region, possibly with both TMH2 and TMH11, and the lipid bilayer. For the NBMPR-bound state, K283R results in stabilisation similar to that seen for wild type, ∆∆*T*_*m*_ 1.2 ± 1.5°C (Figure 2B, Figures 5B, Supplementary Table S2). The retention of stabilisation by NBMPR suggests that, despite this mutation being destabilising for the *apo*-state, the protein is still able to interact with NBMPR in a way that provides wild type-like stabilisation.

#### 3.2.4 Mutation of the ICL6

**FIGURE 6:**
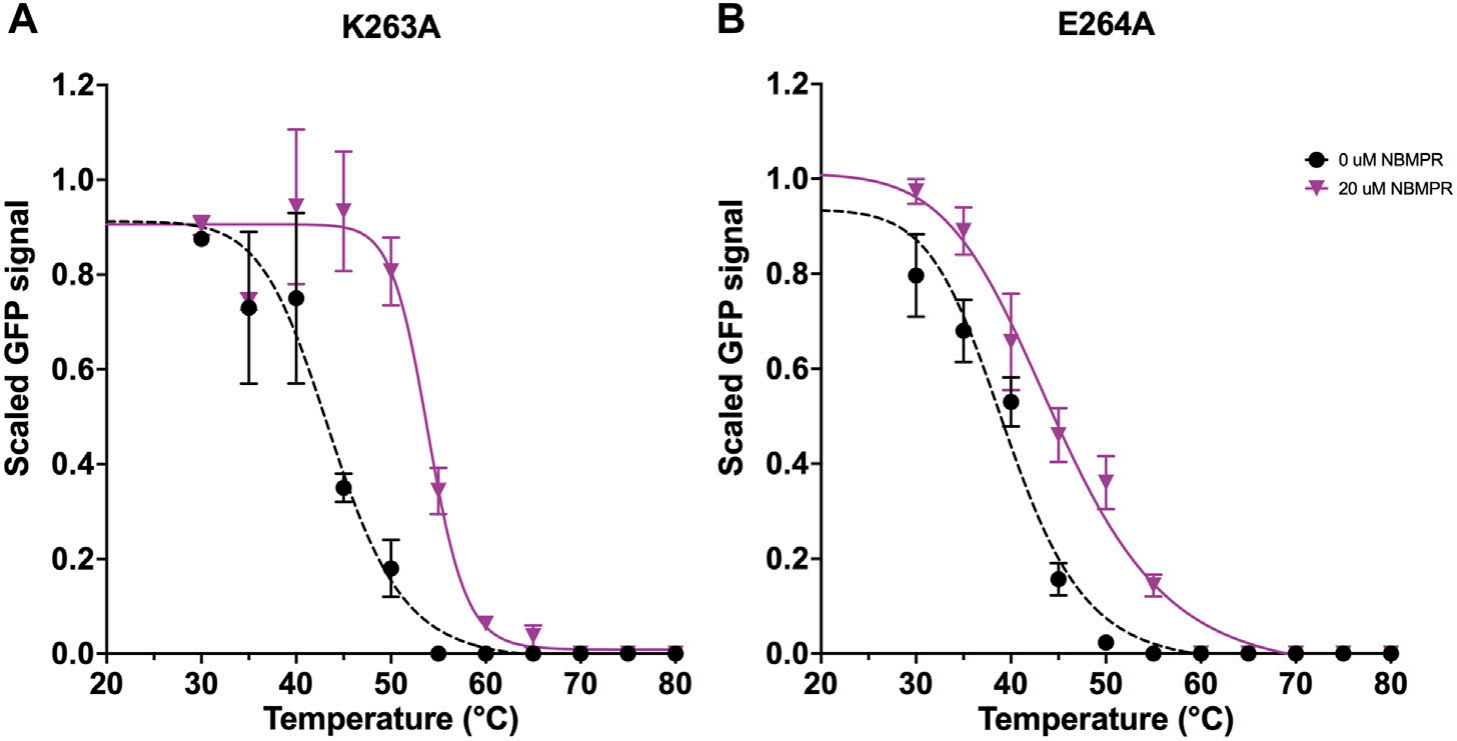
Investigation of variants K263A and E264A stabilisation of hENT1. Data generation, fitting and error analysis performed as detailed for wild type. **(A)** K263A *apo*-state curves were collected as an average of 5 repeats, whereas NBMPR-bound curves were collected as an average of 3 repeats. **(B)** E264A *apo*-state curves were collected as an average of 7 repeats, whereas NBMPR-bound curves were collected as an average of 3 repeats.

K263 and E264 are located at the ICL6. However, in the available structures of hENT1 (PDB: 6OB6 and 6OB7) residues 243–274, which contribute to ICL6, were deleted to generate a construct that was amenable to crystallisation (Wright and Lee, 2019). Mutations K263A and E264A each stabilise the *apo*-state, ∆ *T*_*m*_ of 1.0 ± 0.7°C and 0.7 ± 0.7°C, respectively (Figures 2A, Supplementary Figure S3 and Supplementary Table S1). As the ICL6 is predicted to be largely disordered (Hughes et al., 2015) these variants may stabilise this region in the *apo*-state by reducing conformational flexibility. However, like I282V and K283R, each of these mutations had differing effects on stabilisation of the NBMPR-bound state. While E264A is no more stabilised by NBMPR than wild type, ∆∆ *T*_*m*_ -0.3 ± 1.5°C, K263A is significantly stabilised, ∆∆*T*_*m*_ 5.0 ± 1.7°C (*p* = 0.0001) (Figure 2B, Figure 6, Supplementary Figure S4 and Supplementary Table S2). Additionally, the curve fit to the NBMPR-bound state data of K263A has a steeper hill slope than that of the *apo*-state, and other variant NBMPR-bound curves (Figure 6A). This suggests that K263A may unfold more cooperatively in the NBMPR-bound state. The replacement of the large, charged residue at position 263 with Ala may allow for other NBMPR-bound state stabilising interactions to take place.

#### 3.2.5 Mutation of TM8

**FIGURE 7:**
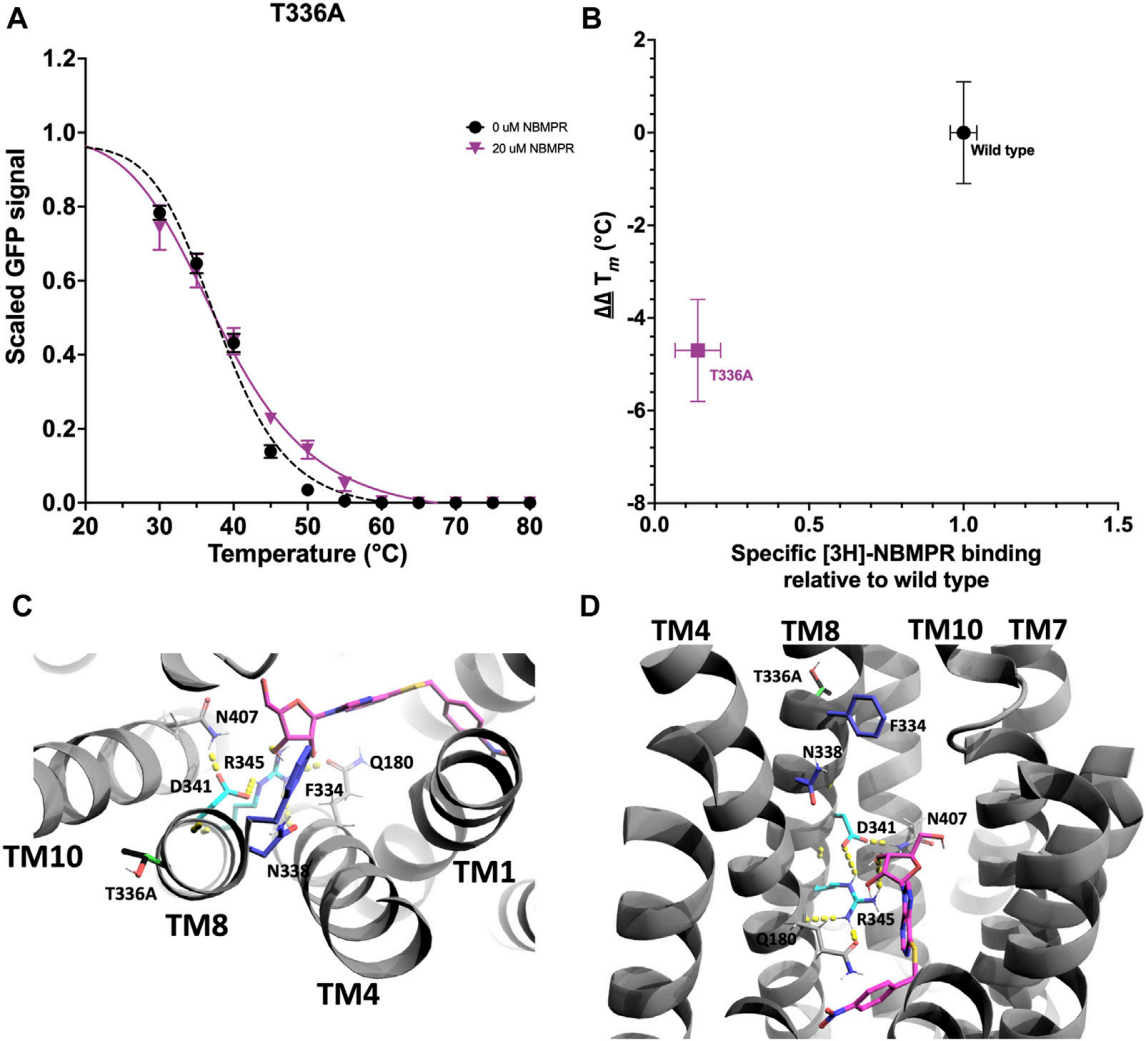
Investigation and rationalisation of variant T336A destabilisation of apo- and NBMPR bound hENT1. Data generation, fitting and error analysis performed as detailed for wild type. **(A)** T336A *apo*-state curves were collected as an average of 12 repeats, whereas NBMPR-bound curves were collected as an average of 6 repeats. **(B)** Scatter plot of hENT1 variant ∆∆ *T*_***m***_ *versus* amount of radiolabelled specific inhibitor [3H]-NBMPR bound in the membrane, relative to wild type. Error bars are representative of error as detailed in “Data-fitting”. **(C)** A top-down and **(D)** A close-up perpendicular view of the central cavity of hENT1 (TM11 is removed in D for clarity). Side chains of residues involved in the surrounding of the ribose moiety of NBMPR (pink) are shown in cyan. Residues previously determined to be important determinants in inhibitor sensitivity are shown in blue. Native T336 is shown in black, and variant T336A in green.

T336 is located at the extracellular region of TM8 and faces towards TM10 and the lipid bilayer (Figure 7C). A number of residues on TM8 which face towards the central cavity have previously been shown to be important determinants in inhibitor sensitivity (Visser et al., 2007), with D341 and R345 specifically being shown to interact with the ribose moiety of NBMPR (Wright and Lee, 2019) (Figure 7D). The mutation T336A has a destabilising effect on the *apo* state, ∆ *T*_*m*_ -1.1 ± 0.6°C, and shows no stabilisation in the presence of NBMPR, ∆∆ *T*_*m*_ -4.7 ± 1.1°C (p = <0.0001) (Figure 2A, Figure 2B, Figures 7A, Supplementary Figure S4, Supplementary Table S1 and Supplementary Table S2). This loss of NBMPR stabilisation in T336A led to further investigations to determine specific [3H]-NBMPR binding. These data suggest that the loss of stabilisation by NBMPR is a result of a specific reduction in binding, with T336A [3H]-NBMPR binding 0.14 times that of wild type (Figure 7B).

## 4 Discussion

### 4.1 A new method for bacmid DNA extraction

To date, the only reported data for bacmid DNA extraction from transfected cell cultures is that of McCarthy & Romanowski, 2008 (McCarthy and Romanowski, 2008). However, this method requires the chloroform extraction of viral particles from the cell-free media. We developed a protocol to make the commercially available kits for the isolation of plasmid DNA from bacterial cultures work for the isolation of bacmid DNA from Sf9 cells. This eliminated the need for both toxic organic solvents and specialist extraction kits. Furthermore, it allows for the validation of bacmid DNA at all steps in the Sf9 expression process. Where baculovirus expression systems require multi-step processes, from DNA transfection to repeat baculovirus infections for increasing the viral titre, the ability to extract bacmid DNA at all steps allows for the confirmation of target integration, screening of cross-contamination, identification of why expression levels have decreased (Garretson et al., 2018), or simply data validation.

### 4.2 The role of TM8 and T336A

hENT1 interactions with the ribose moiety of NBMPR are mediated by D341 and R345 on TM8. D341 is exclusively conserved across mammalian ENTs, and residue 345 is a highly conserved positively charged residue (R/K) (Supplementary Figure S5). In this study we have shown that mutation T336A results in a significant reduction in the binding of NBMPR, with [3H]-NBMPR binding seven times worse than that of wild type hENT1 (Figure 7B). Mammalian orthologues of hENT1 feature a highly conserved polar residue (T/N) at the position equivalent to T336 of TM8 (Supplementary Figure S3). Conversely, the equivalent residue in the NBMPR insensitive isoforms hENT2, hENT3 and hENT4 (and their mammalian orthologues) is a highly conserved hydrophobic residue (L/V). Therefore, the reduction in binding observed in T336A is due to the specific exchange of a polar residue for the NBMPR-insensitive isoform-like hydrophobic residue.

In addition to NBMPR sensitivity, hENT isoforms differ in substrate selectivity. hENT1 has a higher affinity for nucleosides and hENT2 (and hENT3 and hENT4, albeit to a lesser degree) a higher affinity for nucleobases (Baldwin et al., 2004; Young et al., 2008; Young et al., 2013). Interactions with the purine and ribose moiety of NBMPR are suggested to represent interactions with endogenous nucleoside substrates of hENT1. Therefore, interactions with the ribose moiety of nucleosides are predicted to also be mediated by D341 and R345. Interactions with TM10 and/or the lipid bilayer mediated by the residue at position 336 may affect the ability of TM8 to support specific interactions with the ribose moiety of NBMPR and nucleosides via D341 and R345.

### 4.3 Towards understanding the role of hENT1 ICL6

Previous NMR studies have suggested that the ICL6 is unstructured (Reyes et al., 2011a; Reyes et al., 2011b; Aseervatham et al., 2015). However, sequence analysis and computational structural predictions (Jumper et al., 2021; Varadi et al., 2022) suggest that there is an additional short helix at residues 243–256. Nonetheless, despite recent advancements in the field, models produced using currently available computational methods have low to very low confidence in the prediction of this region (Supplementary Figure S6). Therefore, at present, predictions of the orientation and specific conformation of these domains are deemed unreliable.

A major limiting factor in the confident structural prediction of ENTs is the lack of suitable homology models. The distinct‚ significant differences between ENTs and MFS transporters, as demonstrated in the X-ray structures of hENT1 (Supplementary Figure S1) (Wright and Lee, 2019) gives rise to poorly fit and low confidence models. Furthermore, the diversity in the structure and function of loop regions in MFS subfamilies highlights the need for experimentally determined structures. For instance, the MFS sugar porter subfamily, which includes the mammalian glucose transporters (GLUTs) (Deng et al., 2015) and their bacterial homologues XylE (Sun et al., 2012) and GlcP (Iancu et al., 2013), feature an intracellular domain comprised of a series of short helices (the ICH domain) (Supplementary Figure S7B) at the ICL6. This ICH domain directly interacts with TMs and is suggested to act as a latch which secures the closure of the intracellular gating domain in the outward-open conformation (Sun et al., 2012; Iancu et al., 2013; Deng et al., 2015; Nomura et al., 2015). The di-/tripeptide transporter PepT2 (Parker et al., 2021), and the plant nitrate transporter NRT1.1 (Parker and Newstead, 2014), also contain an ICL6 that is predominantly α-helical. However, here the helical loop extends away from the transporter (Supplementary Figure S7C).

The ICL6 bridges the connection between the N- and C-terminal domains of MFS through TM6 and TM7, respectively. TM7 plays a significant role in the mechanism of action in MFS transporters. It is typically present as a discontinuous helix that undergoes rearrangements, such as partial unwinding at the extracellular region, during substrate binding and translocation (Shi, 2013; Deng et al., 2015; Yan, 2015; Zhang et al., 2015; Quistgaard et al., 2016) and, in the sugar porter subfamily, TM7 and the ICH work in tandem to mediate gating interactions at the intracellular domain (Deng et al., 2015). This characteristic discontinuous helix is observed in hENT1 and supports the extracellular gating interactions between P308 and M33 of TM1. However, as only outward-facing inhibitor bound structures of hENT1 are available, it is unknown what rearrangements TM7 may undergo in the transition from *apo*-state to substrate/inhibitor bound and how these rearrangements may influence the ICL6.

In this study we identified variants at the ICL6 (K263A and E264A) and the TM7 (G305A and M306T) that stabilise the *apo*-state of hENT1. Furthermore, we identified that K263A (ICL6) and I282V (TM7) stabilise the inhibitor bound state. We propose that these variants support interactions that contribute to gating at the intracellular face of the NBMPR-bound state, as in the sugar porters. However, without structures of the ICL6, the mechanisms by which this is achieved remain unknown, nor can they be reliably modelled. Additionally, the ICL6 also contains several charged residues that may support interactions with the lipid-bilayer (Reyes et al., 2011a; Parker and Newstead, 2014; Parker et al., 2021). Therefore, where interactions with the lipid-bilayer may contribute to regulation and conformational stabilisation (Valiyaveetil et al., 2002; Phillips et al., 2009; Wang et al., 2022), the study of membrane proteins in a native-like lipid containing environment is essential for understanding native structures and the molecular basis of their mechanism of action.

## 5 Conclusion

hENT1 is proposed to utilise an alternating access mechanism of action. However, distinct differences between ENTs and canonical MFS structures supports the suggestion that ENTs utilise a mechanism of action that is distinct from that of MFS transporters. This is supported through the observation of distinct gating interactions at the intracellular region of hENT1, which are mediated by hydrophobic and highly conserved polar and charged interactions (Wright and Lee, 2019). Furthermore, due to the unique structural features of ENTs there is a lack of suitable theoretical models, and current methods for the computational prediction of ENT structures generates models with overall poor fit and low confidence. The effects of mutations discussed in this study support a role for the ICL6 in the intracellular gating mechanisms of hENT1 (Reyes et al., 2011b; Aseervatham et al., 2015). However, the molecular basis by which this is achieved remains unknown and its mechanisms cannot be properly addressed. This study further highlights the need for experimentally determined full-length hENT1 structures, with the inclusion of key features such as the ICL6, and identifies some mutations that may help in achieving this goal (K263A, N30F and M306T, all of which stabilise more than *wt* in the presence of NBMPR). Moreover, the understanding of the molecular basis of the mechanism of action requires high resolution insights into distinct conformational states and will benefit from their study in more native-like environments (Parker and Newstead, 2014; Chakrapani, 2015; Fowler et al., 2015; Kapsalis et al., 2019; Parker et al., 2021).

## Data Availability

The original contributions presented in the study are included in the article/Supplementary Material, further inquiries can be directed to the corresponding author.
