# Supplementary material for: Novel variants provide differential stabilisation of human equilibrative nucleoside transporter 1 states
Source: Front Mol Biosci. 2022 Nov 8;9:970391. doi: 10.3389/fmolb.2022.970391 (PMC9678926; doi:10.3389/fmolb.2022.970391)
Supplement: Supplementary file 1 [file DataSheet1.docx]

# Supplementary Materials

S1 Fig. Architecture of MFS and ENT transporters. (A) The 12 α-helices of MFS transporters are shown with a rainbow representation, with blue-to-green depicting the N-terminal domain and yellow-to-red the C-terminal domain. Sites of the A-motif, which is essential for the transport activity in many MFS transporters, at the intracellular loop between TM2 and TM3 and/or TM8 and TM9 are highlighted in black. The 12 α-helices are organised into three helical bundle repeats (TM1-3, 4-6, 7-9 and 10-12) and are represented by triangles. (B) A top-down view of a representative MFS uniporter, GLUT3 (PDB: 4ZWB), shows the typical helical arrangement in MFS transporters. (C) The 11 α-helices of ENTs are shown with a rainbow representation. As in MFS, the 11 α-helices are organised into three and two helical bundle repeats (TM1-3, 4-6, 7-9 and 10-11) and are represented by triangles. (D) A top-down of hENT1 (PDB: 6OB6) shows that in hENT1 TM9 is arranged to occupy the space that is shared by both TM9 and TM12 in GLUT3. The overall arrangement of the remaining helices is otherwise similar.

S2 Fig. A representative SDS-PAGE of hENT1 following a ten-temperature challenge. Following incubation at ten-temperature points, (4, 30, 35, 40, 45, 50, 55, 60, 65 and 70 °C), with 0 µM and 20 µM NBMPR, surviving hENT1-linked GFP signal is visualised using fluorescence imaging. hENT1 is present at ~57 kDa, and hENT1 specific signal reduces as temperature increases. The intensity of the protein that remained in solution after the temperature challenge was quantified and data normalised to the 4 °C control.


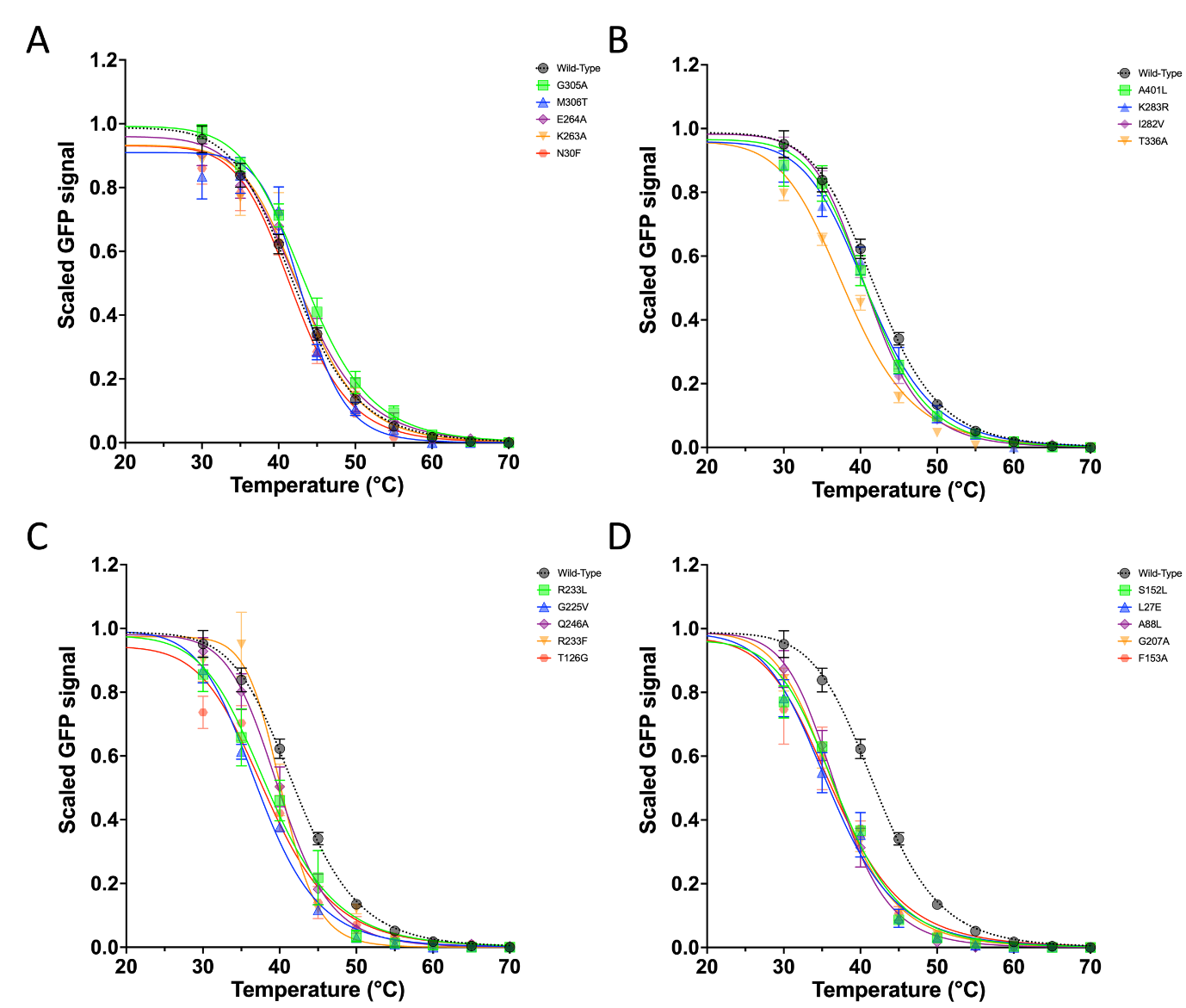


S3 Fig. Ten-temperature stability curves for all hENT1 variants. Protein that remained in solution after challenge in a ten-temperature melting curve was quantified using in-gel GFP fluorescence. The curves are subdivided into the following groups for clarity of presentation: (A) *T_m_* = 42.0 – 43.5 °C, (B) *T_m_* = 40.8 – 41.1 °C, (C) *T_m_* = 37.2 – 40.3 °C, and (D) *T_m_* = 36.1 – 37.1 °C. hENT1 variant curves were initially collected in biological triplicates, as detailed in “Expression cultures and solubilisation”. However, following variant validation through bacmid DNA extraction, PCR amplification and Sanger sequencing, the number of repeats increased for several variants. Wild type curves were collected as an average of 15 repeats, whereas variant curves were collected as an average of 3-12 repeats. Data in each curve are normalised to the intensity of the sample incubated on ice. Error bars are representative of SEM. Data were fit with a four-parameter dose-response curve (variable slope) by non-linear least-squares fitting in GraphPad Prism 9.0.





S4 Fig. T_m_ values of hENT1 variants in the *apo* and NBMPR bound state. (A) No statistical significance was observed for any variants that were identified as stabilising when using the 0.6 °C cut-off, as calculated by average SEM ∆*T_m_* (B) Statistically significant differences are indicated for K263A (p = 0.0001) and T336A (p = <0.0001) with *** and ****, respectively. A401L and R233F were omitted from NBMPR analysis due to issues faced with cell culture contamination.


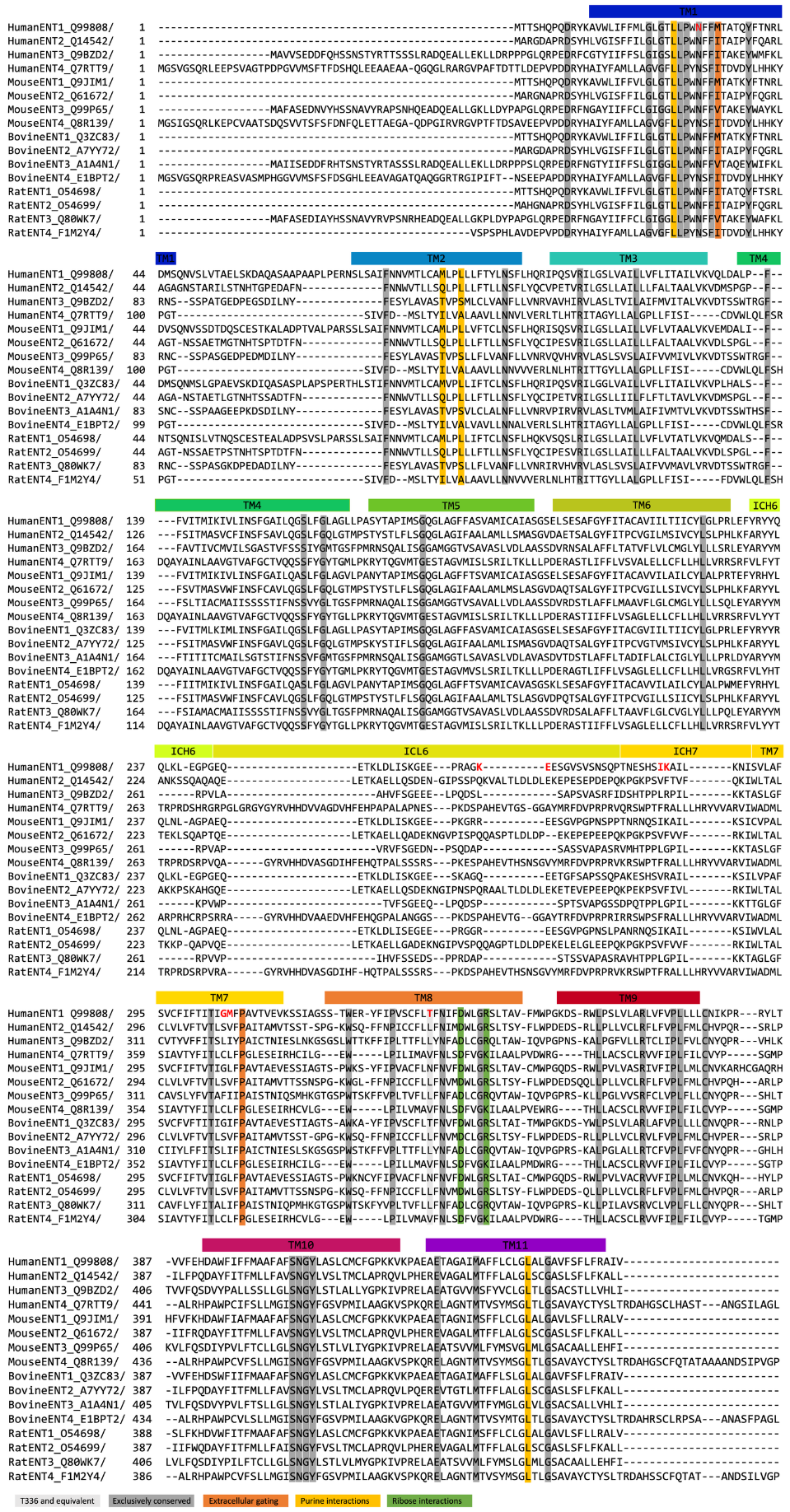


S5 Fig. Multiple sequence alignment of select mammalian ENTs with UniProt identifiers. Residues mutated in this study are displayed in red, T336 and equivalent residues are highlighted in light grey. Residues that are exclusively conserved are highlighted in dark grey. Residues involved in extracellular gating interactions are highlighted in orange. Residues discussed involved in interactions with the purine and ribose moiety of NBMPR are highlighted in yellow and green, respectively.


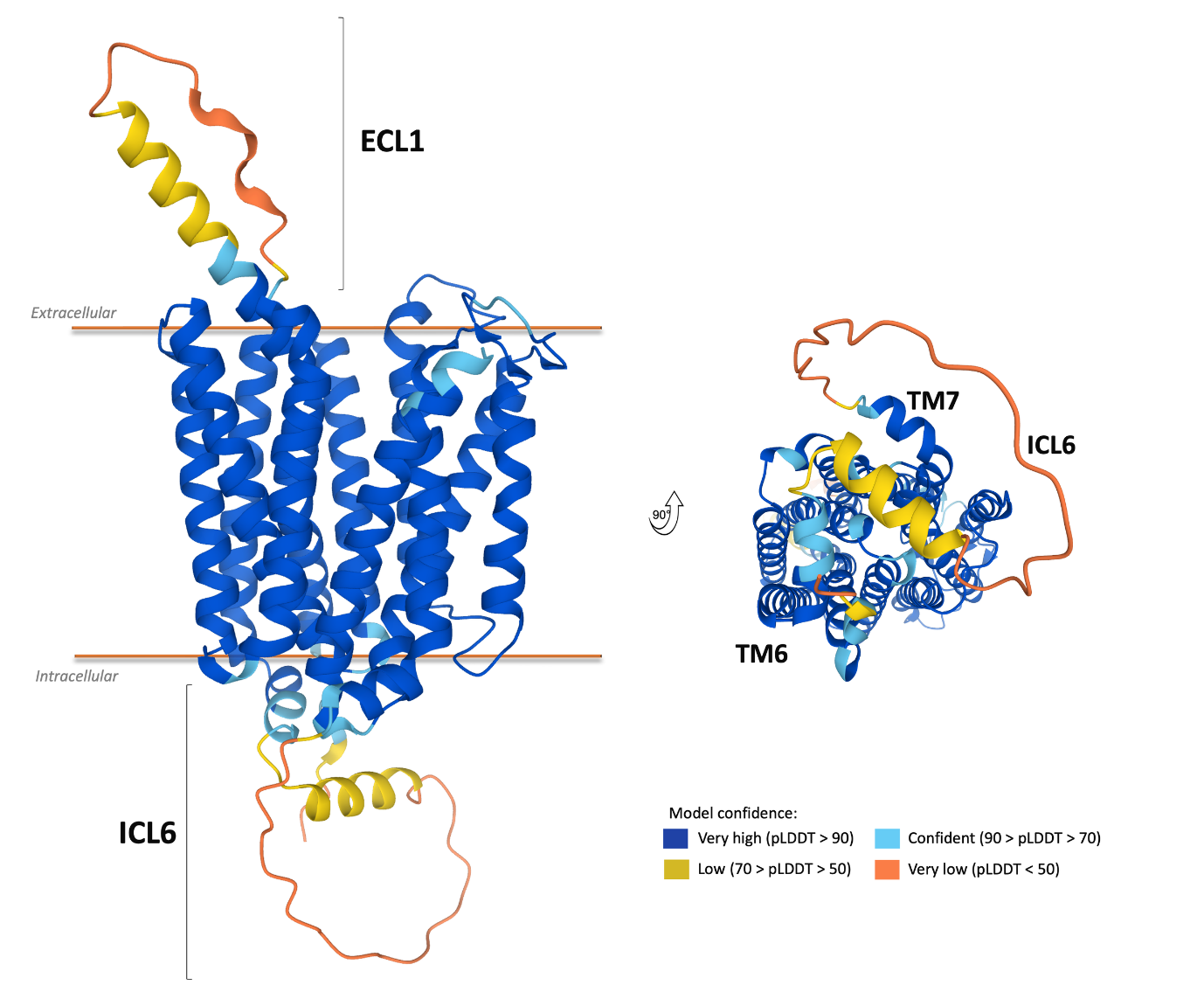


S6 Fig. Structural prediction of full-length hENT1. The ECL1 and ICL6 are absent from the structures of hENT1 in complex with inhibitors NBMPR (PDB: 6OB6) and dilazep (PDB: 6OB7). A structural prediction of the full-length wild type hENT1 was obtained from the Alphafold Protein Structure Database (Varadi et al., 2022). Model confidence is ranked, on a per-residue confidence score (pLDDT), as very high (dark blue, pLDDT > 90), confident (light blue, pLDDT >70), low (yellow, pLDDT > 50) and very low (orange, pLDDT < 50).


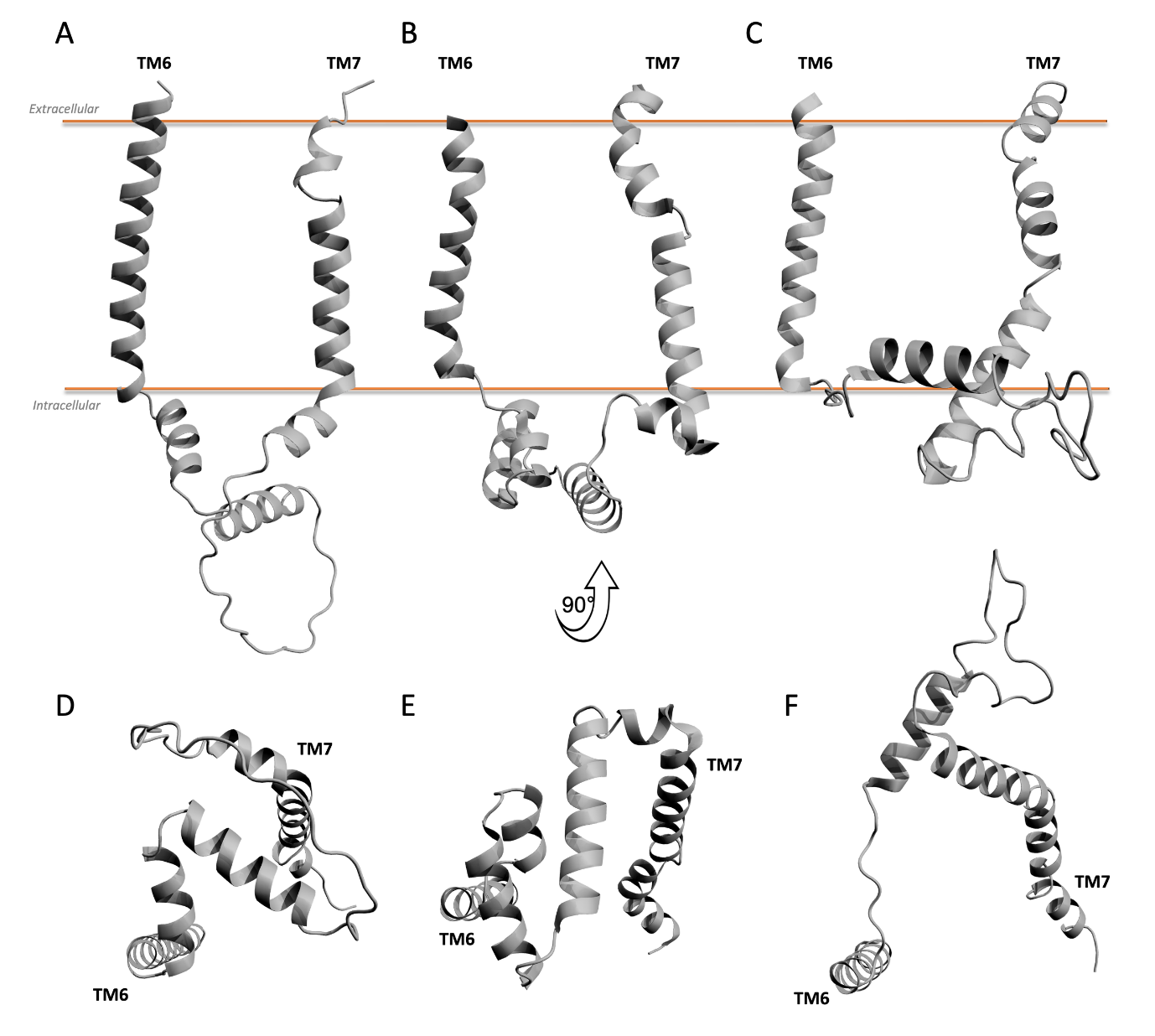


S7 Fig. Representations of differing ICL6 domains of MFS members. Representative structures of the TM6, ICL and TM7 of (A, D) Alphafold (Jumper et al., 2021) predicted full-length wild type hENT1, (B, E) the mammalian sugar transporters, Glut3 (PDB: 4ZW9) and (C, F) the mammalian peptide transporter PepT2 (PDB: 7NQK).

S1 Table Results of ten-temperature melting analysis of apo-hENT1 variants with identities validated by DNA sequencing.

| Variant | *T_m_* (°C)^a^ | *T_m_* error (±°C)^b^ | ⍙ *T_m_* (°C)^c^ | ⍙ *T_m_* error (±°C)^d^ | Repeats (n) | Status | Significance  (P value)^e^ |
| --- | --- | --- | --- | --- | --- | --- | --- |
| G305A | 43.5 | 0.4 | 1.5 | 0.5 | 8 | Stabilising | ns (p = 0.0794) |
| M306T | 43.2 | 0.5 | 1.1 | 0.6 | 5 | Stabilising | ns (p = 0.6440) |
| K263A | 43.0 | 0.6 | 1.0 | 0.7 | 5 | Stabilising | ns (p = 0.7620) |
| E264A | 42.7 | 0.6 | 0.7 | 0.7 | 7 | Stabilising | ns (p = 0.9502) |
| Wild Type | 42.0 | 0.3 | 0.0 | 0.5 | 15 | N/A | N/A |
| N30F | 42.0 | 0.5 | 0.0 | 0.6 | 10 | Neutral | ns (p = 0.9997) |
| K283R | 41.1 | 0.5 | -0.9 | 0.6 | 12 | Destabilising | ns (p = 0.3553) |
| A401L | 41.0 | 0.5 | -1.0 | 0.6 | 9 | Destabilising | ns (p = 0.3059) |
| T336A | 40.9 | 0.5 | -1.1 | 0.6 | 12 | Destabilising | ns (p = 0.2046) |
| I282V | 40.8 | 0.3 | -1.2 | 0.5 | 12 | Destabilising | ns (p = 0.0744) |
| R233F | 40.3 | 0.5 | -1.8 | 0.6 | 3 | Destabilising | ns (p = 0.1341) |
| Q246A | 40.0 | 0.4 | -2.0 | 0.6 | 6 | Destabilising | ** (p = 0.0047) |
| R233L | 38.6 | 0.8 | -3.4 | 0.9 | 6 | Destabilising | **** (p = <0.0001) |
| T126G | 38.4 | 0.8 | -3.7 | 0.8 | 3 | Destabilising | *** (p = 0.0001) |
| G225V | 37.2 | 0.3 | -4.8 | 0.4 | 3 | Destabilising | **** (p = <0.0001) |
| S152L | 37.1 | 0.4 | -4.9 | 0.6 | 3 | Destabilising | **** (p = <0.0001 |
| A88L | 36.9 | 0.4 | -5.1 | 0.5 | 3 | Destabilising | **** (p = <0.0001) |
| G207A | 36.7 | 0.3 | -5.3 | 0.5 | 3 | Destabilising | **** (p = <0.0001) |
| F153A | 36.5 | 0.8 | -5.5 | 0.9 | 3 | Destabilising | **** (p = <0.0001) |
| L27E | 36.1 | 0.6 | -5.9 | 0.7 | 6 | Destabilising | **** (p = <0.0001) |

aAverage *T_m_* was calculated from individual *T_m_* estimated for each individual repeat by fitting with a four-parameter dose-response curve (variable slope) by non-linear least-squares fitting in GraphPad Prism 9.0

bStandard error of the mean (SEM) shown.

c⍙ *T_m_* represents variant relative to wild type.

dError calculated and propagated as detailed in Methods and materials: Data fitting and statistical analysis section.

e Statistical analysis performed on Tm data using ordinary one-way ANOVA with a Dunnet follow-up test for multiple comparisons

S2 Table ∆ Tm results of melting analysis of NBMPR-bound hENT1 variants.

| Variant | ⍙ *T_m_* (°C) | ⍙ *T_m_* error (±°C)^a^ | Repeats (n) |
| --- | --- | --- | --- |
| K263A | 10.0 | 1.5 | 3 |
| N30F | 9.9 | 2.0 | 3 |
| I282V | 8.0 | 1.6 | 3 |
| M306T | 6.7 | 1.5 | 3 |
| K283R | 6.2 | 1.7 | 3 |
| Wild Type | 5.0 | 0.8 | 3 |
| G305A | 4.0 | 0.7 | 3 |
| E264A | 4.7 | 1.3 | 3 |
| T336A | 0.3 | 0.8 | 6 |

aError calculated and propagated as detailed in Methods and materials: Data fitting and statistical analysis section.
